# Associated Biochemical and Hematological Markers in COVID-19 Severity Prediction

**DOI:** 10.1155/2023/6216528

**Published:** 2023-10-19

**Authors:** Anit Lamichhane, Sushant Pokhrel, Tika Bahadur Thapa, Ojaswee Shrestha, Anuradha Kadel, Govardhan Joshi, Sudip Khanal

**Affiliations:** ^1^Department of Laboratory Medicine, Manmohan Memorial Institute of Health Sciences, Kathmandu, Nepal; ^2^Department of Pathology, Sumeru Hospital Pvt Ltd., Lalitpur, Nepal; ^3^Department of Public Health, Manmohan Memorial Institute of Health Sciences, Kathmandu, Nepal

## Abstract

**Background:**

The global threat of COVID-19 has created the need for researchers to investigate the disease's progression, especially through the use of biomarkers to inform interventions. This study aims to assess the correlations of laboratory parameters to determine the severity of COVID-19 infection.

**Methods:**

This study was conducted among 191 COVID-19 patients in Sumeru Hospital, Lalitpur, Nepal. According to their clinical outcomes, these patients were divided into severe and nonsevere groups. Inflammatory markers such as LDH, D-dimer, CRP, ferritin, complete blood cell count, liver function tests, and renal function tests were performed. Binary logistic regression analysis determined relative risk factors associated with severe COVID-19. The area under the curve (AUC) was calculated with ROC curves to assess the potential predictive value of risk factors.

**Results:**

Out of 191 patients, 38 (19.8%) subjects died due to COVID-19 complications, while 156 (81.7%) survived and were discharged from hospital. The COVID-19 severity was found in patients with older age and comorbidities such as CKD, HTN, DM, COPD, and pneumonia. Parameters such as d-dimer, CRP, LDH, SGPT, neutrophil, lymphocyte count, and LMR were significant independent risk factors for the severity of the disease. The AUC was highest for d-dimer (AUC = 0.874) with a sensitivity of 82.2% and specificity of 81.2%. Similarly, the cut-off values for other factors were age >54.5 years, D-dimer >0.91 ng/ml, CRP >82.4 mg/dl, neutrophil >78.5%, LDH >600 U/L, and SGPT >35.5 U/L, respectively.

**Conclusion:**

Endorsement of biochemical and hematological parameters with their cut-off values also aids in predicting COVID-19 severity. The biomarkers such as D-dimer, CRP levels, LDH, ALT, and neutrophil count could be used to predict disease severity. So, timely analysis of these markers might allow early prediction of disease progression.

## 1. Introduction

Coronavirus disease 2019 (COVID-19) caused by severe acute respiratory syndrome coronavirus-2 has become a major global health problem leading to pandemic outbreaks [1]. Most studies illustrated that elevated ferritin, CRP, and d-dimer were associated with mortality risk [2]. Early severity prediction can help to provide quality treatment, which might enhance the survival rate by lowering mortality [3]. For the prognosis of the infection, laboratory parameters such as the hematological profile and the inflammatory index have a significant predictive value. Laboratory features such as leukocytosis, lymphopenia, neutrophilia, NLR, PLR, CRP, LDH, D-dimer, ferritin level, and liver enzymes are significantly associated with the severity of the disease [4]. With an increase in the severity of the disease, lymphopenia is encountered at a higher rate in the nonsurvival patients compared to the survivors [5]. In COVID-19, due to viral activity, hematological and inflammatory marker changes occur that cause damage to liver tissue leading to a rise in ALT, AST, and LDH activity [5].

A study by Shang et al. states that hemodialysis patients possess a greater risk of COVID-19 infection [6]. Conversely, comorbidities such as diabetes, hypertension, immunodeficiency, and cardiovascular disease are considered risk factors for COVID-19 infection whereas acute respiratory distress syndrome (ARDS), septic shock, acute kidney injury, cardiac injury, and multiorgan failure are identified as complications of COVID-19 disease [7].

A better understanding of early prognostic clinical laboratory parameters helped to save many lives by enabling timely intervention and better resource allocation since ICU capacity is limited in most of the countries [8]. Hence, this study aims to find the clinical and laboratory biomarkers associated with COVID-19 severity.

## 2. Materials and Methods

Patients diagnosed with COVID-19 by quantitative reverse transcription PCR (qRT-PCR) and admitted to Sumeru Hospital Pvt. Ltd., Lalitpur, Nepal, from March 2021 to October 2021, were included in this study.

### 2.1. Exclusion and Inclusion Criteria

Patients with COVID-19 infection were further verified by positive reverse transcription polymerase chain reaction (RT-PCR), and clinical symptoms were included in the study. Patients with asymptomatic conditions and duplicate samples were excluded from the study.

All patients above 18 years, fulfilling the criteria mentioned previously, were given informed and written consent and recorded their demographic data, comorbidities, and laboratory parameters using standard performa.

### 2.2. Experimental Protocol

Blood sample was collected in different vacutainer system. Whole blood was collected in an EDTA vacutainer and analyzed hematological parameters: complete blood cell count (CBC) including hemoglobin, red blood cell (RBC), packed cell volume (PCV), platelet count, white blood cell (WBC), and differential count using a fully automated blood cell counter (Sysmex XN-350). Trisodium citrate plasma was collected to analyze the D-dimer test using an immunofluorescent analyzer (iChroma II). Serum was separated to analyze different biochemical tests such as random blood sugar (RBS), lactate dehydrogenase (LDH), renal function tests including urea, creatinine, sodium, and potassium, and liver function tests including total bilirubin (TB), direct bilirubin (DB), alanine transferase (ALT), aspartate transferase (AST), and alkaline phosphatase (ALP) using fully automated biochemistry analyzer (Erba XL 200). Ferritin was analyzed from fully automated CLIA (Roche Cobas e411), and C-reactive protein (CRP) was analyzed by an immunofluorescent analyzer (iChroma II). Daily internal quality control and quarterly external quality control were conducted to validate tests.

COVID-19 diagnosis and clinical classification were made from the new coronavirus pneumonia diagnosis and treatment plan (Trial Version 7) established by the People's Republic of China's National Health Commission as follows:

1. Mild, with few symptoms and no evidence of pneumonia on imaging
2. Moderate, with fever and signs of pneumonia on imaging
3. Severe, with any of the following: the following symptoms of respiratory distress were observed: (a) respiratory rate 30 beats/min; (b) oxygen saturation 93%; (c) arterial blood oxygen partial pressure 300 mmHg; and (d) pulmonary imaging revealed a lesion that had advanced by more than 50% in 24–48 hours
4. A critical condition, one of the following: a respiratory failure that necessitates mechanical ventilation, shock, and a need for ICU admission due to multiple organ failure [9]

Furthermore, we categorized the COVID-19-infected patients into two categories as mild and moderate or severe according to the above-mentioned criteria.

### 2.3. Statistical Analysis

The data were analyzed in IBM SPSS (IBM Corp. Released 2019. IBM SPSS Statistics for Windows, Version 26.0. Armonk, NY: IBM Corp). Normally distributed data were expressed as mean and standard deviation and compared by independent sample *t*-test. Nonnormally distributed data were presented as median and interquartile range and compared by the Mann–Whitney U test. The Chi-square test compared categorical variables. Pearson's correlation was used to determine the correlation between severity and variables. All variables were subjected to univariate logistic regression, and odds ratios were calculated between nonsevere and severe groups, with a 95% confidence interval. Variables were included in binary logistic regression if the corresponding *P* value was less than 0.05. Binary logistic regression analysis was used to determine relative risk factors associated with severe COVID-19. The area under the curve (AUC) was calculated with ROC curves to assess the cut-off values of potential predictive factors.

## 3. Results

Out of 191 patients, 101 patients belong to the mild symptomatic group and 90 to the severe group. The average age of patients was found to be 54.9 ± 17.9 years. The mean age of the severe group (60.31 ± 17.35) was significantly higher (*P* < 0.001) than that of the mild group (50.08 ± 17.10) (Table 1).

Based on the clinical history, the prevalence of comorbidities such as CKD, HTN, DM, COPD, anemia, and pneumonia was significantly higher in the severe population (*P* < 0.001, *P* = 0.034, *P* = 0.049, *P* < 0.001, *P* = 0.013, and *P* = 0.005, respectively). The demographic, clinical, and laboratory parameters of the patients are presented in Table 1.

Among hematological parameters, total WBC count (*P* < 0.001), neutrophil count (*P* < 0.001), lymphocyte count (*P* < 0.001), NLR (*P* = 0.001), and PLR (*P* < 0.001) were significantly higher, whereas RBC count (*P* = 0.001), LMR (*P* = 0.017), and LCR (*P* < 0.001) were lower among moderate and severe cases as compared to mild infection.

Similarly, biochemical parameters such as urea (*P* < 0.001), creatinine (*P* = 0.001), ALT (*P* = 0.001), AST (*P* < 0.001), ferritin (*P* < 0.001), LDH (*P* < 0.001), CRP (*P* < 0.001), and D-dimer (*P* < 0.001) were significantly higher in moderate and severe patients as compared to mild cases (Table 1).

In the univariate analysis, risk factors associated with disease severity were age, NLR, LMR, PLR, LCR, ferritin, LDH, CRP, D-dimer, RBC count, Hb, WBC count, neutrophilia, lymphopenia, PCV, urea, creatinine, and aminotransferases. Variables that significantly affect the disease progression revealed by univariate analysis were entered into multivariate logistic regression analysis. Variables such as age, LMR, LDH, CRP, D-dimer, neutrophil, lymphocyte count, and AST remained the independent risk factors for the severity of the disease (Table 2).

**Figure 1:**
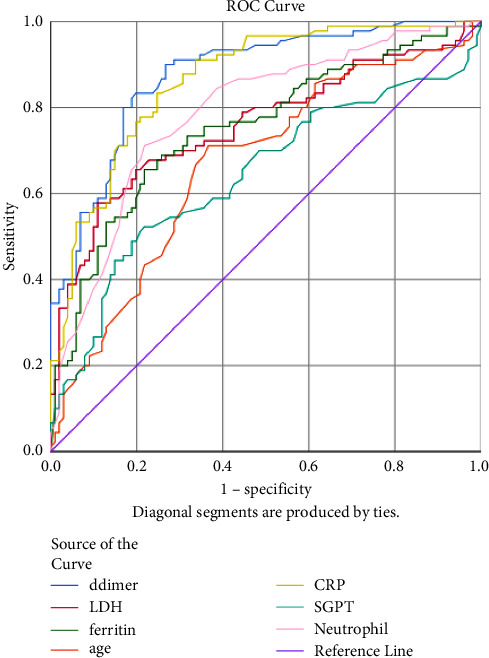
Receiving operating characteristics for generating cut-off values of d-dimer, LDH, ferritin, age, CRP, SGPT, and neutrophil for the severity of COVID-19 infection.

The AUROC, standard error, and 95% CI of each parameter are shown in Table 3, and the cut-off value was estimated from Figure 1. The area under the curve was highest for D-dimer (AUC = 0.874, 95% CI = 0.824–0.924, *P* < 0.001), and the best cut-off point was 0.91 ng/ml, with a sensitivity of 82.2% and specificity of 81.2%. Similarly, the ROC curve of CRP (AUC = 0.857, 95% CI = 0.803–0.910, *P* < 0.001) suggested the best cut-off point of 82.4 mg/dl with a sensitivity of 82% and specificity of 75.2%. Similarly, the cut-off values for age, neutrophil, LDH, ALT, and ferritin were 54.5 years, 78.5%, 600 IU/L, 35.5 IU/L, and 43.5 ng/ml, respectively, for the severity prediction (Figure 1).

## 4. Discussion

The pandemic outbreak of COVID-19 infection worldwide has created a heavy burden on healthcare services. Mortality is the major issue in dealing with this pandemic, and early identification of severe and critical cases is essential to minimize the mortality and improve the recovery rate [10]. The fatality rate was 4.3–15% among hospitalized COVID-19 patients [6]. Therefore, to determine the severity of COVID-19 infection, it is necessary to ascertain the early predictors that could help clinicians to identify the early stage of infection for timely diagnosis and treatment [11].

We examined the demographic and laboratory characteristics of 191 COVID-19-infected individuals in this study, and 38 (19.8%) died afterward. In our study, the population of the moderate and severe groups was significantly higher in age as compared to that of the mild group, with the best cut-off point by ROC being 54.4 years, which is similar to the study of Wang et al. [12]. A multicenter study showed that in the older age group, the risk of mortality increased by 18% [13]. COVID-19 patients suffering from comorbidities were at high risk for mortality [13]. Several studies suggested that proper consideration must be provided to comorbidity [14]. In our study, the patients suffering from hypertension, CKD, DM, COPD, pneumonia, and anemia were highly severe, similar to a study by Yilmaz et al. [7, 13]. Similarly, a higher mortality was observed in the COVID-19 patients suffering from CKD, which is in accordance with the study of Posso et al. Furthermore, studies suggested that COVID-19 infection and human immunological response could lead to kidney illness [15].

Among the laboratory findings, Hb, total WBC count, neutrophil count, lymphocyte count, urea, creatinine, potassium, ALT, AST, Ferritin, LDH, CRP, D-dimer, NLR, and PLR were found to be significantly higher; however, RBC count, LMR, and LCR were lower among the moderate or severe group of the population as compared to the mild group, which is similar with several studies [3, 14, 16]. In severe cases, neutrophilia might be due to the activation of neutrophils as an immunological response to the virus [14, 16]. This result is in support of the study of Yang et al. reported higher NLR and PLR and lower LMR and LCR in the severe group [17]. NLR and PLR were positively correlated with critical illness, whereas LMR and LCR were negatively correlated.

In this study, D-dimer is one of the major risk factors associated with fatal outcomes in COVID-19 patients. D-dimer increases in the disseminated intravascular coagulation are considered an early stage pulmonary intravascular coagulopathy. A study suggested severe thrombosis in tiny capillaries and microvasculature in the lung tissue of COVID-19 patients, indicating that D-dimer is a significant prognostic indicator in individuals with probable infection and sepsis [18].

CRP, an acute-phase protein, is independently associated with critical illness in COVID-19. Our study showed higher CRP levels in the severe group of patients with an odds ratio of 1.021 in multivariate logistic regression, which is in accordance with the study of Sharifpour et al. [19]. CRP being an independent prognostic factor may facilitate risk stratification and prognostication. In this study, CRP and d-dimer were found to be independent risk factors associated with fatal outcomes, which is similar to the study of Liu et al. and Wang et al. [12, 20].

Similarly, based on ROC analysis among severe/moderate and mild populations, the cut-off values of different variables such as age is >54.5 years, D-dimer is >0.91 ng/ml, CRP is >82.4 mg/dl, neutrophil is >78.5%, LDH is >600 U/L, and SGPT is >35.5 U/L for severity prediction. It indicates the progression of the disease to a critical case which must be observed for the prevention of COVID-19 complications [12]. These findings suggest that routine monitoring of these parameters is useful for improving the early diagnosis of critical COVID-19 and establishing an accurate therapeutic strategy [21].

However, the study was limited to a single center, and the sample size was smaller. In addition, the patient's disease state, clinical symptoms, onset, and exposure history were not considered. Furthermore, radiological diagnoses such as CT scan and other laboratory indicators associated with severity, such as cytokines and procalcitonin, were not measured.

## 5. Conclusion

This study revealed that elder age, D-dimer, CRP levels, LDH, ALT, and neutrophil count were independent severity risk factors as compared to the case of mild patients. Evaluation of these factors could aid in detecting disease progression. Early medical care and assistance for these high-risk patients may help to minimize the disease's fatality rate.

### 5.1. Clinical Significance

Early diagnosis and monitoring of the disease progression play crucial roles in the management of COVID-19 complications. [22] Screening of biochemical and hematological biomarkers, primarily D-dimer, CRP, LDH, ALT, and neutrophil count helps triaging COVID-19-infected elderly individuals early in the disease's course.

## Figures and Tables

**Table 1:** Sociodemographic and other laboratory investigations with the prognosis of COVID-19.

Parameters	Mild (*N* = 101)	Moderate/severe (*N* = 90)	*P* value
Demographic characteristics			
Gender			
Male	61 (60.4%)	53 (58.9%)	
Female	40 (39.6%)	37 (41.1%)	
Age $\left(\bar{x}\pm \sigma \right)$ (years)	50.08 ± 17.10	60.31 ± 17.35	**0.001**
Comorbidities			
CKD	18 (17.8%)	40 (44.4%)	**0.001**
HTN	11 (10.9%)	20 (22.2%)	**0.034**
DM	32 (31.7%)	41 (45.6%)	**0.049**
COPD	2 (2.0%)	20 (22.2%)	**0.001**
Anemia	17 (16.8%)	29 (32.2%)	
Pneumonia	2 (2.0%)	11 (12.2%)	**0.005**
Hematological parameters			
Hb (gm/dl)	12.7 ± 2.7	11.5 ± 2.5	**0.001**
Total WBC count (cells/cumm)	6418.8 ± 2586.9	9177.8 ± 4829.9	**0.001**
Neutrophil count (%)	70.7 ± 12.5	82.5 ± 9.7	**0.001**
Lymphocyte count (%)	24.6 ± 11.6	13.9 ± 8.4	**0.001**
Monocyte count (%)	3.5 ± 2.2	3.5 ± 10.4	0.965
Eosinophil count (%)	1.3 ± 1.2	1.3 ± 4.3	0.959
Platelets count (cells/cumm)	216603 ± 83486	217577 ± 82925	0.936
PCV (%)	38.1 ± 7.9	31.9 ± 7.5	0.14
RBC count (cells/cumm)	4.3 ± 0.9	3.96 ± 0.9	**0.001**
NLR	3.3 (1.9–5)	6.9 (4.4–11.4)	**0.001**
LMR	7.3 (5–11)	5.7 (3.1–9.5)	**0.017**
PLR	8750 (6242–12398)	18437 (10269–28380)	**0.001**
LCR	0.6 (0.2–2.5)	0.1 (0.056–0.25)	**0.001**
Biochemical parameters			
Urea (mg/dl)	26 (20–39)	49 (28–99.2)	**0.001**
Creatinine (mg/dl)	1.0 (0.85–1.3)	1.3 (1–4.3)	**0.001**
Sodium (mEq/L)	136 ± 4.6	135.7 ± 6.4	0.631
Potassium (mEq/L)	3.9 ± 0.5	4.1 ± 0.7	**0.03**
ALT (U/L)	30 (20.5–45)	50 (27.7–77)	**0.001**
AST (U/L)	32 (24–50)	47.5 (32–70.5)	**0.001**
ALP (U/L)	84.9 ± 39.9	90.2 ± 33.3	0.325
Total bilirubin (mg/dl)	0.6 (0.4–0.8)	0.6 (0.4–0.8)	0.454
Direct bilirubin (mg/dl)	0.2 (0.2–0.3)	0.2 (0.2–0.3)	0.247
Ferritin (ng/ml)	409 (268.3–623.5)	945 (474.8–1530)	**0.001**
LDH (U/L)	500 (384–662.5)	870 (533.5–1200.2)	**0.001**
CRP (mg/dl)	39 (13–84.4)	136.5 (93.7–188.5)	**0.001**
D-dimer (ng/ml)	0.45 (0.2–0.8)	1.95 (1.0–5.7)	**0.001**

CKD: chronic kidney disease; HTN: hypertension; DM: diabetes mellitus; COPD: chronic obstructive pulmonary disease; Hb: hemoglobin; PCV: packed cell volume; ALT: alanine transferase; AST: aspartate transferase; ALP: alkaline phosphatase; LDH: lactate dehydrogenase; CRP: C-reactive protein; NLR: neutrophil-to-lymphocyte ratio; LMR: lymphocyte-to-monocyte ratio; PLR: platelet-to-lymphocyte ratio; LCR: lymphocyte-to-CRP ratio. *P* value indicates the level of significance.

**Table 2:** Univariate and multivariate logistic regression analyses of risk factors associated with severe COVID-19.

Parameters	Univariate				Multivariate			
	*β*	SE	95% CI	*P*	*β*	SE	95% CI	*P* value
Age	1.035	0.009	1.017–1.035	0.001^*∗*^	1.031	0.014	1.003–1.060	0.030^*∗*^
Hb	0.819	0.059	0.729–0.919	0.001^*∗*^				
RBC	0.57	0.168	0.410–0.793	0.001^*∗*^				
PCV	0.945	0.020	0.909–0.982	0.004^*∗*^				
TC	1.000	0.000	1.0–1.0	0.001^*∗*^				
Neutrophil	1.100	0.017	1.065–1.137	0.001^*∗*^	0.844	0.085	0.714–0.997	0.047^*∗*^
Lymphocyte	0.895	0.020	0.861–0.930	0.001^*∗*^	0.828	0.095	0.688–0.9980	0.048^*∗*^
NLR	1.296	0.049	1.176–1.47	0.001^*∗*^				
LMR	0.939	0.029	0.887–0.995	0.032^*∗*^	1.107	0.052	1.000–1.226	0.050
LCR	0.722	0.123	0.567–0.919	0.001^*∗*^				
Ferritin	1.002	0.000	1.001–1.003	0.001^*∗*^				
LDH	1.003	0.001	1.002–1.004	0.001^*∗*^	1.001	0.001	1.000–1.003	0.050
CRP	1.027	0.004	1.019–1.034	0.001^*∗*^	1.021	0.008	1.012–1.043	0.001^*∗*^
D-dimer	2.891	0.213	1.905–4.387	0.001^*∗*^	1.830	0.209	1.214–2.758	0.004^*∗*^
Urea	1.012	0.003	1.006–1.019	0.001^*∗*^				
Creatinine	1.225	0.062	1.085–1.383	0.001^*∗*^				
Potassium	1.785	0.239	1.117–2.851	0.015^*∗*^				
ALT	1.018	0.005	1.008–1.028	0.001^*∗*^	1.026	0.008	1.010–1.041	0.001^*∗*^
AST	1.022	0.006	1.010–1.035	0.001^*∗*^				
ALP	1.003	0.004	0.95–1.010	0.498				

Hb: hemoglobin; PCV: packed cell volume; TC: total leukocyte count; ALT: alanine transferase; AST: aspartate transferase; ALP: alkaline phosphatase; LDH: lactate dehydrogenase; CRP-C: reactive protein; NLR: neutrophil-to-lymphocyte ratio; LMR: lymphocyte-to-monocyte ratio; PLR: platelet-to-lymphocyte ratio; LCR: lymphocyte-to-CRP ratio. *P* value indicates the level of significance.

**Table 3:** Predictive efficacy of the severe COVID-19 risk model and early predictors.

Test variables	AUC	SE	95% CI	Cut-off point	Sensitivity (%)	Specificity (%)	*P* value
D-dimer (ng/ml)	0.874	0.021	0.824–0.924	0.91	82.2	81.2	0.001^*∗*^
Age (years)	0.656	0.031	0.577–0.736	54.5	71.1	63.4	0.001^*∗*^
CRP (mg/dl)	0.857	0.047	0.803–0.910	82.4	82	75.2	0.001^*∗*^
Neutrophil (%)	0.774	0.043	0.706–0.842	78.5	73.3	72.3	0.001^*∗*^
LDH (U/L)	0.762	0.023	0.691–0.833	600	71.1	66.4	0.001^*∗*^
ALT (U/L)	0.648	0.012	0.566–0.730	35.5	60.0	54.0	0.001^*∗*^
Ferritin (ng/ml)	0.750	0.036	0.591–0.745	43.5	80.0	59.4	0.001^*∗*^

ALT: alanine transferase; AST: aspartate transferase; ALP: alkaline phosphatase; LDH: lactate dehydrogenase; AUC: area under curve; CI: confidence interval. *P* value indicates the level of significance.

## Data Availability

The data used to support the findings are included in the manuscript, if needed please contact us for correspondence.
